# Validation Study of BioSystems^®^ Y15 Histamine Dehydrogenase Kit for the Detection of Histamine in Fish and Fishery Products: AOAC *Performance Tested Method*^SM^ 072001

**DOI:** 10.1093/jaoacint/qsaa139

**Published:** 2020-10-14

**Authors:** Andreu Tobeña, Sabina Dueñas, Mercè Boix

**Affiliations:** BioSystems S.A., Costa Brava 30, Barcelona, Catalonia, Spain, 08030

## Abstract

**Background:**

BioSystems has developed a histamine kit for automated procedure, through the use of a Y15 analyzer, for quantification of histamine in fish and fishery products.

**Objective:**

Validate the method under the specific guidelines of the AOAC Research Institute *Performance Tested Method*^SM^ (PTM) program.

**Method:**

Samples are extracted with boiling water. The enzymatic method is based on histamine dehydrogenase that catalyzes the oxidation of histamine in the presence of an electron mediator that reduces a dye that is measured at 420 nm. The increase of absorbance is proportional to the histamine concentration. Dispensing of reagents and sample, absorbance readings, calibration, and calculation of results are performed automatically in the analyzer BioSystems Y15.

**Results:**

The linearity ranges from 0 to 200 mg/kg (r^2^ > 0.99). The LOQ is 10 mg/kg in all the matrixes. Recoveries range from 75 to 107% at concentrations from 5 to 200 mg/kg, with repeatability precision values between 0.8 and 5.5%. Comparisons with the HPLC reference method shows a good correlation. Cross-reactivity of the assay is negligible for all biogenic amines tested except for agmatine (6.3%). Product consistency was verified by validating lot-to-lot variations and variations within the same lot. Shelf life was verified by real-time stability testing during 40 months at 2–8°C. No differences in histamine detection were observed in robustness testing, in which minor changes are introduced to the assay protocol.

**Conclusions:**

The automated, simple, and rapid BioSystems Y15 Histamine Dehydrogenase Kit has been successfully validated.

**Highlights:**

The method is qualified for PTM certification No. 072001.

## General Information

Histamine and other biogenic amines are generated in improperly stored raw fish by enzymatic conversion of free histidine and other amino acids. Decarboxylase producing Gram-negative enteric bacteria are primarily responsible for the formation of histamine in raw fish and fishery products. Improper storage conditions (time/temperature) are the main reason for bacterial growth. Consumption of such mishandled fish can lead to histamine fish poisoning, also termed scombroid poisoning. The symptoms are similar to those associated with seafood allergies (1).

Histamine fish poisoning is an allergy-like form of food poisoning that continues to be a major problem in seafood safety. The symptoms are variable and include peppery or metallic taste, oral numbness, headache, dizziness, palpitations, rapid and weak pulse (low blood pressure), difficulty in swallowing, and thirst. Noteworthy as allergy-like are symptoms such as hives, rash, flushing, and facial swelling. Symptoms involving the central nervous system such as anxiety are less frequently observed. Less specific symptoms such as nausea, vomiting, abdominal cramps, and diarrhea are also experienced (2).

The FAO/WHO Codex Alimentarius as well as European Union (EU) and United States Food and Drug Administration (FDA) legislation have therefore set maximum limits for histamine in fish and fishery products. Regulation (EC) No 2073/2005 limits the content of histamine in fishery products from fish species associated with a high amount of histidine to between 100–200 mg/kg, and in fishery products which have undergone enzyme maturation treatment in brine, manufactured from fish species associated with a high amount of histidine between 200–400 mg/kg. Codex Alimentarius limits histamine to 200 mg/kg for species of *Clupeidae*, *Scombridae*, *Scombresocidae*, *Pomatomidae*, and *Coryphaenedae* families, whereas the FDA has set a guidance level of 50 mg/kg histamine in an edible portion of fish (3).

## Principle

**Figure 1.**
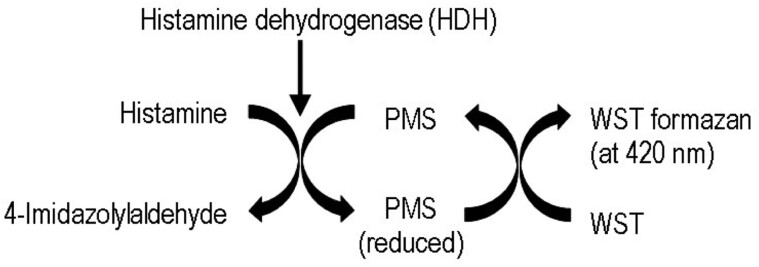
Schematic illustration of the colorimetric reaction principle.

The enzyme histamine dehydrogenase (HDH) catalyzes the oxidation of histamine to 4-amidazolylaldehyde in the presence of 1-methoxy-5-methylphenazinium methyl sulfate (PMS), a photochemically stable electron mediator and a water-soluble tetrazolium salt (WST). When WST is reduced, the corresponding formazan dye is formed and can be measured at 420 nm. The increase of absorbance is proportional to the histamine concentration (Figure 1). Once the sample has been extracted, the necessary actions to measure histamine (dispensing of reagents and sample, absorbance readings, calibration, and calculation of results based on the weight of the sample) are performed automatically in the random access analyzer BioSystems Y15.

## Scope of Method

- *Analyte(s).*—Histamine [2-(4-Imidazolyl)-ethylamine]; CAS Registry No. 51-45-6.
- *Matrixes.*—Fresh tuna, frozen tuna, water-canned tuna, oil-canned tuna, raw salmon, raw sardines, oil-canned sardines, semi-preserved anchovy fillets.
- *Summary of validated performance claims*.—Based on internal validation study:
  1. *Precision*.—Recovery within 80–110%.
  2. *Accuracy*.—Less than 10% RSD_r_ of averages at different concentrations tested in all matrixes (above LOQ).
  3. *Selectivity*.—Less than 6.5% cross-reactivity with agmatine and negligible (<0.2%) with other closely related compounds.
  4. *LOQ*.—LOQ is 10 mg/kg for fish and fish products.
  5. *Range of quantitation*.—This assay has a range of quantitation between 10 and 200 mg/kg without additional dilution. The range can be extended through further dilution with water.

### Intended Users

Technical staff of food analysis laboratories in the fishery sector, whether primary or processing.

### Definitions

- *Linearity*.—Ability of the assay to obtain responses that are directly proportional to the concentration of the analyte in the sample.
- *Selectivity*.—Ability of the method to detect the analyte without interference from matrix or other components of similar behavior.
- *Recovery*.—Fraction or percentage of the analyte that is recovered when the test sample is analyzed using the entire method. There are two types of recovery: *(1)* Total recovery based on recovery of the native plus added analyte, and *(2)* marginal recovery based only on the added analyte (the native analyte is subtracted from both the numerator and denominator). It is expressed as the ratio of the mean candidate method result to the true value, expressed as a percentage, (concentration of fortified samples/concentration of unfortified samples – concentration of analyte added to the test sample) × 100. Unless indicated, the term “recovery” in the text and in the results refers to “total recovery.”
- *Accuracy*.—Ratio of the mean candidate method result to the reference method result, expressed as a percentage, (mean_cand_/mean_ref_) × 100.
- *Bias*.—Difference between the candidate method mean result and the true value, mean_cand_ – known spike.
- *LOD*.—Lowest analyte concentration that can be detected in the sample but not necessarily quantitated under the experimentally established conditions. The following formula is used to calculate the LOD:
  $$\text{LOD}=\frac{{\bar{X}}_{0}+3.3(s_{b})}{1-1.65m}$$
  where ${\bar{X}}_{0}$ = the mean analytical value of the non-spiked matrix, *S_b_* = the *y*-intercept of the line, and *m* = the slope of the line.
- *LOQ*.—Lowest analyte that can be determined with acceptable precision and accuracy in a sample under the conditions of the method used (confirmed experimentally). Estimated by the 3 × LOD formula, then checked by testing 10 replicates in each matrix.
- *Precision*.—Degree of agreement between independent test results obtained under predefined conditions. Precision is usually expressed as imprecision by calculating the calculated relative standard deviations and Horwitz relative standard deviations.
- *Robustness*.—Susceptibility of an analytical method to variations in the experimental conditions, such as the type of matrix analyzed and which can be expressed as a list of sample materials, analytes, sample storage, environmental or preparation conditions under which the method can be applied as specified, or with certain minor modifications.

## Materials and Methods

### Test Kit Information

- *Kit name*.—HISTAMINE.
- *Catalog No*.—12829.
- *Ordering information*.—BioSystems S.A., Costa Brava 30, 08030 Barcelona (Spain).

### Test Kit Components

- *A*.—Reagent. 2 × 40 mL. Buffer 25 mmol/L, PMS, WST. pH = 9.0.
- *B*.—Reagent. 1 × 20 mL. Buffer 25 mmol/L, HDH. pH = 9.0.
- *S1*.—Standard. 1 × 5 mL. Histamine 2.5 mg/L. Aqueous primary standard.
- *S2*.—Standard. 1 × 5 mL. Histamine 5.0 mg/L. Aqueous primary standard.
- *S3*.—Standard. 1 × 5 mL. Histamine 10.0 mg/L. Aqueous primary standard.
- *S4.*—Standard. 1 × 5 mL. Histamine 20.0 mg/L. Aqueous primary standard.
- *S5*.—Standard. 1 × 5 mL. Histamine 33.3 mg/L. Aqueous primary standard.

### Apparatus

- *Analytical balance*.
- *Blender*.
- *Homogenizer*.
- *Hot plate stirrer*.—Suitable for boiling water.
- *Magnetic stirring bar*.
- *Vortex mixer*.
- *Centrifuge*.
- *BioSystems Y15 Analyzer.*

### Additional Supplies and Reagents

- *Distilled water*.
- *Polypropylene tube*.—50 mL.
- *Graduated cylinder*.—125 mL.
- *Syringe filter*.—For example, Whatman Cat. No. 6884-2510.
- *Adjustable pipettes*.—Capable of delivering 20–200, 100–1000, and 500–5000 μL.
- *Microcentrifuge tube*.—1.5 mL (Eppendorf or similar).

### Reference Materials

All reference and quality control materials and proficiency test samples were purchased from FAPAS (York, United Kingdom; http://www.fapas.com).

- *Canned fish*.—Histamine in Canned Fish Quality Control Material (High Levels) T27132QC.
- *Canned fish*.—Histamine in Canned Fish Reference Material (Low Levels) TET040RM.
- *Canned fish*.—Food Analysis Proficiency Assessment Scheme 27243 March–May 2019.
- *Canned fish*.—Food Analysis Proficiency Assessment Scheme 27189 November 2016–January 2017.
- *Canned fish*.—Food Analysis Proficiency Assessment Scheme 27253 August–September 2019.

### Standard Solutions

The calibrators provided with the test kit are prepared by weighing the pure analyte (histamine dihydrochloride—H7250 purity >99% from Sigma-Aldrich) after appropriate drying of the material in a scale in a suitable state of calibration. The analyte is dissolved avoiding any loss and is made up to the final volume in a volumetric flask or by weighing.

### Safety Precautions

Reagents should be stored at 2–8°C. Components are stable once opened until the expiry date stated in the label, if they are stored well closed and care is taken to prevent contamination during their use. Do not use the kit past the expiration date.

The manipulation of the histamine test kit must be done according to the hazard and precaution indications (Reagent B: WARNING: H317: May cause an allergic skin reaction. P302 + P352: IF ON SKIN: Wash with plenty of soap and water. P333 + P313: If skin irritation or rash occurs: Get medical advice/attention). For further warnings and precautions, see the product safety data sheet.

### General Preparation

Reagents and standards are provided ready to use. Components are stable once opened until the expiry date stated in the label, if they are stored well closed and care is taken to prevent contamination during their use.

Histamine adsorbs on glass surfaces. Therefore, the use of glassware should be avoided during sample preparation (4).

### Sample Preparation

1. For sampling and sample homogenization, follow AOAC *Official Method^SM^* **937.07** (5).
2. Accurately weigh approximately 5 g of homogenized sample and add 25 mL of distilled water.
3. Shake until the sample is homogeneously suspended.
4. Incubate the mix for 20 min in a boiling water bath (100°C), stirring periodically.
5. Let stand to room temperature.
6. Centrifuge for 10 min, at least at 2000 *g* or transfer a part (e.g., 1.5 mL) to a microcentrifuge tube and centrifuge at high speed (>10000 *g*) for 2 min. Use the supernatant.
7. If a layer of fat is observed after centrifugation, take the supernatant through the fat layer, pipette into another microcentrifuge tube and centrifuge again. If the supernatant is turbid, filter with a syringe filter (e.g., Whatman Cat. No. 6884-2510).
8. The histamine in the supernatant is stable for at least 1 day at 15–25°C, 7 days at 2–8°C, or 3 months at −20°C.

### Analysis

The BioSystems Y15 Histamine is an autoanalyzer consisting of a three-axis Cartesian robotic arm, a ceramic piston pump, racks for sample tray, reagent tray, and a reaction rotor housed in a 37°C chamber. The arm holds a sampling syringe needle for drawing sample extract and reagents, dispensing them into the reaction well, kept in the reaction rotor. The dispensing speed and the geometry of the reaction well create a homogeneous mixture and initiate the chemical reaction. The dye reagent changes from colorless to a blue color. The absorbance of the color is measured at 420 nm and is proportional to the concentration of the histamine in the sample extract. The instrument is controlled by a computer installed with specialized software. The computer screen displays both real time status of the analysis and the results of the analysis.

### Software Calculations

**Figure 2.**
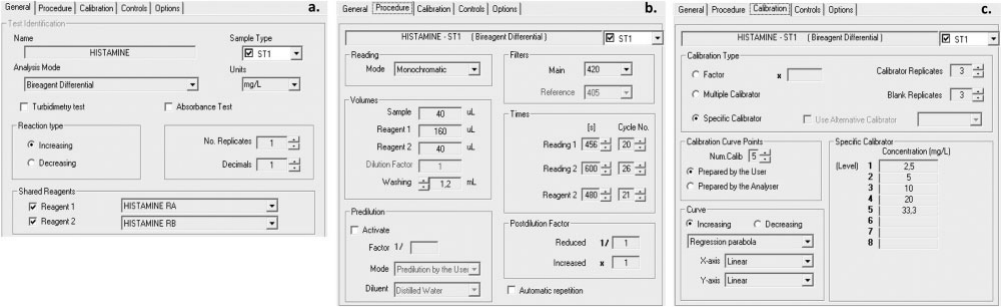
Assay Parameters for histamine in BioSystems Y15 software, (a) General (e.g., analysis mode/ reaction type), (b) Procedure (e.g., reagent volumes, wavelength, and measuring cycles), (c) Calibration (e.g., 5-point calibration and mathematic adjustment by regression parabola).

Y15 is supplied with easy-to-use software to facilitate laboratory routine. The histamine test is already programmed in the software (there is no need to change any parameter). All parameters are shown in different tabs (Figure 2).

### Operating Procedure

#### Calibration procedure

**Figure 3.**
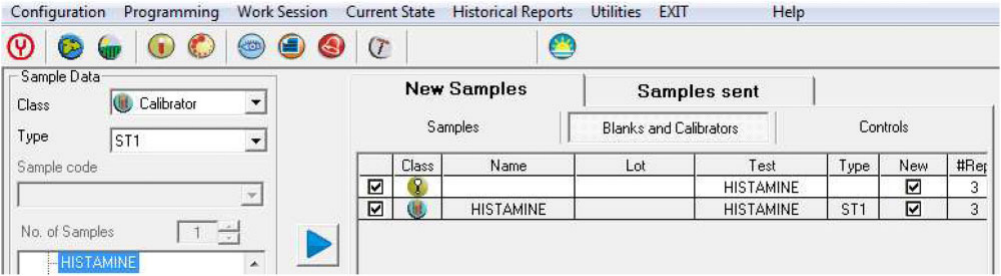
Work session for calibrating samples.

**Figure 4.**
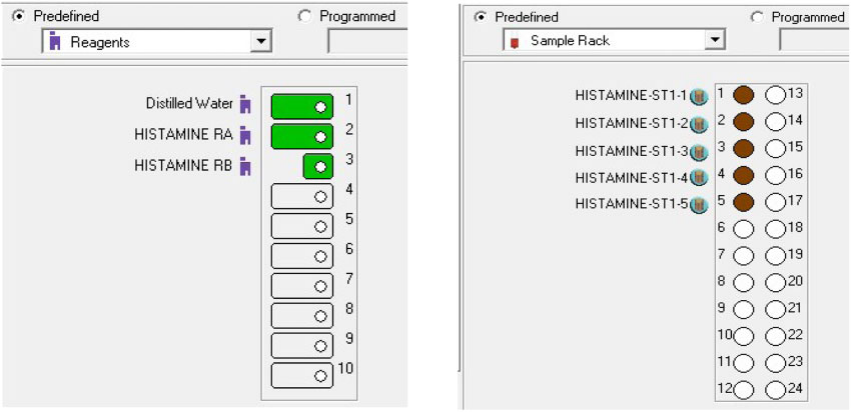
Positioning of reagents and calibrators.

Select “Work Session – New Sample” and in “Class” select “Calibrator.” Check in the list for “Histamine” and click to send the “Blanks & Calibrators” to analyze for the very first time (Figure 3). Position all the calibrators in the “Sample Rack” and Reagents A and B in the “Reagents rack” (Figure 4). Use the “Auto-reagents” and “Auto-sample” buttons.

**Figure 5.**
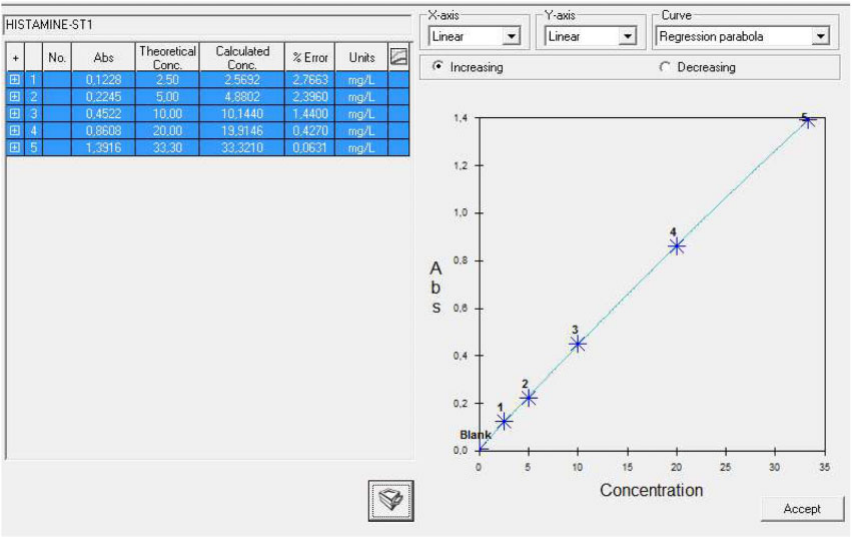
Calibration results.

Once calibrated, the computer software obtains test results using the parabola equation y=ax^2^ + bx + c where y is the absorbance and x is the concentration. Parameters *a*, *b* and *c* are found by using the least squares method. The software will use this curve once inverted to calculate the concentration of the samples from the measured absorbance of their corresponding reactions (Figure 5).

#### Sample analysis

**Figure 6.**
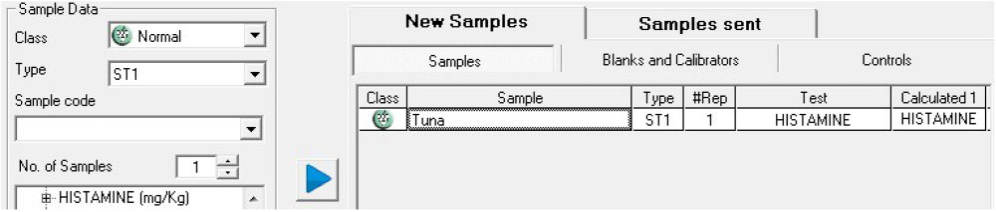
Sample positioning.

**Figure 7.**
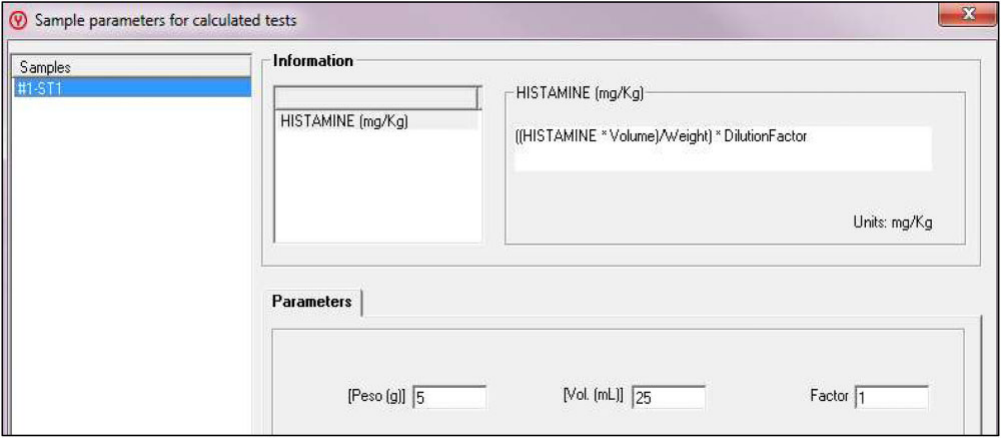
Introduction of sample weight.

**Figure 8.**
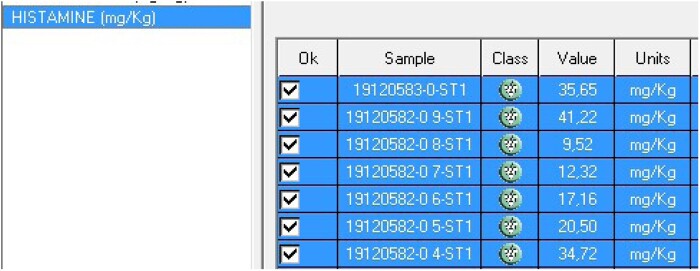
Histamine sample results in mg/kg.

Select “Work Session → New Sample,” press the test “Histamine (mg/kg)” and introduce the number of samples. Position the samples, name them, and press the “Position” button (Figure 6). Position the reagents and samples with “Auto-reagents” and “Auto-samples” buttons. Send them to analyze (“Accept” and “Start”). Introduce the exact weight of each sample by using the “Scale” button in the main screen (Figure 7). Check results by clicking “Current State → Results” (Figure 8). Results can be printed, exported, or saved in “Historical Reports”.

## Validation Study

This validation study was conducted under the AOAC Research Institute *Performance Tested Method*^SM^ program (6). The BioSystems Y15 Histamine automated method was validated for fish (raw tuna, water-canned tuna, oil-canned tuna, raw sardines, oil-canned sardines, raw salmon) and for canned salted fish (semi-preserved anchovy fillets). The manual procedure for this method was not included in the current validation. The studies by the method developer included linearity, LOD, LOQ, bias, recovery and precision, selectivity, lot-to-lot consistency, stability, and robustness. The studies by the independent laboratory included LOD, LOQ, bias, recovery, and precision.

## Method Developer Studies

### Linearity Study

A quantitative analytical method is linear when there is a mathematically verified straight-line relationship between the observed values and the true concentrations of the analyte.

#### Design and methodology

The BioSystems Y15 was calibrated according to the proposed calibration procedure of the Histamine method. Histamine dihydrochloride (H7250 Purity >99% from Sigma-Aldrich) was dissolved in water to make a 1000 mg/L stock standard. The stock standard was further diluted to make the concentrations regularly distributed over the studied range of values (0, 10, 50, 100, 150, and 200 mg/kg). The reference materials were measured five times under reproducibility conditions according to the written method. Appropriate regression statistics and residuals (difference between observed *y* value and calculated *y* value predicted by the straight line, for each *x* value) were calculated and plotted (Table 1). Random distribution of residuals about zero confirms linearity. Systematic trends indicate nonlinearity.

**Table 1.** Results of linearity study of the calibrators

Calibrator value, mg/L	Measured value, mg/L					Results			
	Replica 1	Replica 2	Replica 3	Replica 4	Replica 5	Mean value, mg/l	S_r_	RSD_r_, %	Bias, mg/L
0	0.0	–0.2	–0.2	–0.2	0.0	–0.1	0.12	N/A	–0.1
10	10.0	10.0	9.9	9.8	10.0	9.9	0.11	1.08	–0.1
50	50.9	50.6	50.7	50.7	50.7	50.7	0.11	0.22	0.7
100	99.5	99.2	99.5	99.6	99.7	99.5	0.20	0.20	–0.5
150	150.8	150.3	150.3	150.9	150.1	150.5	0.35	0.24	0.5
200	199.2	198.3	199.6	198.6	199.8	199.1	0.64	0.32	–0.9

N/A: Not Applicable.

#### Results

**Figure 9.**
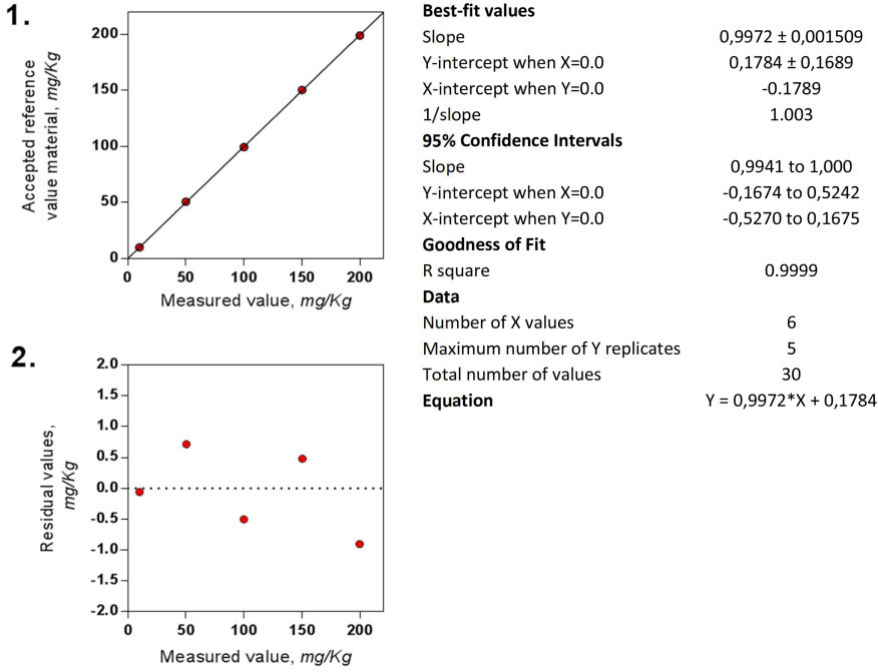
Representation and data of the (1) measured values against accepted values of the reference materials and (2) residual values against the measured values.

Linearity and residual graphs and the corresponding statics are presented in Figure 9. The results show a linear behavior from 0 to 200 mg/kg, with regression statistics that meet the established response/concentration factor criteria.

### Selectivity

#### Design and methodology

Analytical selectivity relates to the extent to which the method can be used to determine the analyte in mixtures or matrixes without interferences from other components of similar behavior. Potential interfering compounds were selected based on structural similarity to histamine and also associated with seafood decomposition. Interfering compounds were tested in each matrix at 1000 mg/kg in the presence and absence of histamine (0 and 25 mg/kg) to evaluate both positive and negative interferences. Potential interfering compounds are described in Table 2 and all were purchased from Sigma-Aldrich.

**Table 2.** Interfering substances used in selectivity studies (Sigma-Aldrich)

Interfering substances	Product	Cat. No.
L-Histidine	L-Histidine, ReagentPlus TM, 99%	H8000
L-Phenylalanine	L-Phenylalanine BioUltra, >99.0% (NT)	78019
Putrescine	Putrescine dihydrochloride	P7505
Cadaverine	1,5-Diaminopentane, 95%	D22606
Tryptamine	Tryptamine, >97.0%	193747
Tyramine	Tyramine hydrochloride	T2879
Methylhistamine	1-Methylhistamine dihydrochloride	M4910
L-Tyrosine	L-Tyrosine disodium salt hydrate	T1145
Anserine	L-Anserine nitrate salt, hydroxyl	A1131
Carnosine	L-Carnosine crystalline	C9625
Agmatine	Agmatine sulfate salt >97% (powder)	A7127

The samples used for selectivity studies were raw tuna, water-canned tuna, oil-canned tuna, raw sardines, oil-canned sardines, and semi-preserved anchovy fillets. Two portions of each sample were weighed (5 g portions) and spiked with the stock solution (5 mg/mL) of each potential interfering compounds to obtain the final concentration of 1000 mg/kg in the matrix. In addition, one of the 5 g portions of each matrix was spiked with the stock solution to obtain the final concentration of 25 mg/kg histamine in the matrix. The samples were extracted and measured with the BioSystems Y15 according to the steps in the Analysis section.

#### Results

The results obtained for the selectivity study are shown in Table 3. Of all the possible interfering substances studied, only agmatine had a positive interference with all matrixes (between 4.6% for semi-preserved anchovy fillets and 6.3% for raw tuna). It is described in scientific papers (7) that the enzyme HDH from *Rhizobium sp*. presents cross-reactivity with agmatine as it oxidizes slightly.

**Table 3.** Results of selectivity of enzyme HDH in matrixes

Histamine mg/kg	Interferent^a^	Raw tuna			Water-canned tuna			Oil-canned tuna			Raw sardine			Oil-canned sardine			Semi-preserved anchovy fillets		
		Result, mg/kg	Δ^b^	%^c^	Result, mg/kg	Δ	%	Result, mg/kg	Δ	%	Result, mg/kg	Δ	%	Result, mg/kg	Δ	%	Result, mg/kg	Δ	%
0	No interferent added	3.4	N/A	N/A	1.6	N/A	N/A	4.2	N/A	N/A	5.1	N/A	N/A	4.8	N/A	N/A	3.6	N/A	N/A
	Methylhistamine	3.3	0.0	0.0	1.5	0.0	0.0	4.3	0.0	0.0	5.0	0.0	0.0	4.6	0.0	0.0	3.7	0.0	0.0
	Tyramine	3.4	0.1	0.0	1.8	0.3	0.0	4.2	–0.1	0.0	4.8	–0.2	0.0	4.2	–0.4	0.0	4.0	0.3	0.0
	L-Phenylalanine	3.5	0.2	0.0	1.7	0.2	0.0	4.4	0.1	0.0	4.9	–0.1	0.0	4.5	–0.1	0.0	3.9	0.2	0.0
	L-Histidine	3.2	–0.1	0.0	1.8	0.3	0.0	4.2	–0.1	0.0	4.9	–0.1	0.0	4.4	–0.2	0.0	3.8	0.1	0.0
	L-Tyrosine	3.3	0.0	0.0	1.8	0.3	0.0	4.1	–0.2	0.0	5.0	0.0	0.0	4.4	–0.2	0.0	3.8	0.1	0.0
	Tryptamine	3.4	0.1	0.0	1.6	0.1	0.0	4.5	0.2	0.0	5.1	0.1	0.0	4.7	0.1	0.0	3.6	–0.1	0.0
	Cadaverine	3.4	0.1	0.0	1.6	0.1	0.0	4.5	0.2	0.0	4.8	–0.2	0.0	4.8	0.2	0.0	3.7	0.0	0.0
	Putrescine	5.2	1.9	0.2	3.3	1.8	0.2	5.8	1.5	0.2	4.9	–0.1	0.0	4.3	–0.3	0.0	4.8	1.1	0.1
	Anserine	3.4	0.1	0.0	1.7	0.2	0.0	4.4	0.1	0.0	4.9	–0.1	0.0	4.6	0.0	0.0	4.1	0.4	0.0
	Carnosine	3.5	0.2	0.0	1.7	0.2	0.0	4.4	0.1	0.0	5.0	0.0	0.0	4.5	–0.1	0.0	4.0	0.3	0.0
	Agmatine	65.8	62.5	6.3	60.2	58.7	5.9	52.7	48.4	4.8	56.2	51.2	5.1	61.2	56.6	5.7	51.6	47.9	4.8
25	No interferent added	28.2	N/A	N/A	25.6	N/A	N/A	28.9	N/A	N/A	28.3	N/A	N/A	30.1	N/A	N/A	28.5	N/A	N/A
	Methylhistamine	27.5	0.0	0.0	25.3	0.0	0.0	29.1	0.0	0.0	28.6	0.0	0.0	30.2	0.0	0.0	29.0	0.0	0.0
	Tyramine	26.7	–0.8	–0.1	25.2	–0.1	0.0	28.2	–0.9	–0.1	29.7	1.1	0.1	30.5	0.3	0.0	29.2	0.2	0.0
	L-Phenylalanine	28.6	1.1	0.1	27.5	2.2	0.2	28.6	–0.5	–0.1	29.5	0.9	0.1	29.9	–0.3	0.0	28.9	–0.1	0.0
	L-Histidine	28.1	0.6	0.1	28.5	3.2	0.3	29.4	0.3	0.0	28.0	–0.6	–0.1	30.5	0.3	0.0	28.7	–0.3	0.0
	L-Tyrosine	27.0	–0.5	–0.1	26.3	1.0	0.1	28.7	–0.4	0.0	29.1	0.5	0.1	30.9	0.7	0.1	29.1	0.1	0.0
	Tryptamine	27.2	–0.3	0.0	29.1	3.8	0.4	29.3	0.2	0.0	28.9	0.3	0.0	31.2	1.0	0.1	28.1	–0.9	–0.1
	Cadaverine	28.5	1.0	0.1	28.7	3.4	0.3	29.0	–0.1	0.0	27.9	–0.7	–0.1	29.9	–0.3	0.0	29.5	0.5	0.1
	Putrescine	28.7	1.2	0.1	29.4	4.1	0.4	29.8	0.7	0.1	29.0	0.4	0.0	30.8	0.6	0.1	30.7	1.7	0.2
	Anserine	28.2	0.7	0.1	26.1	0.8	0.1	29.9	0.8	0.1	28.9	0.3	0.0	30.9	0.7	0.1	29.9	0.9	0.1
	Carnosine	28.4	0.9	0.1	26.0	0.7	0.1	28.7	–0.4	0.0	29.1	0.5	0.1	31.0	0.8	0.1	28.7	–0.3	0.0
	Agmatine	90.2	62.7	6.3	84.7	59.4	5.9	77.1	48.0	4.8	79.6	51.0	5.1	85.9	55.7	5.6	75.1	46.1	4.6

a 1000 mg/kg.

b Δ = mg/Kg (interferent) – mg/kg (no interferent added).

c % = [mg/kg (no interferent added)/mg/Kg (interferent)] × 100.

N/A: Not Applicable.

Histamine, tyramine, cadaverine, and putrescine are the most common biogenic amines formed in fish, fishery products, and seafood, so that interference caused by agmatine is of little importance in the measurement of histamine (8).

### Matrix Study: Accuracy and Precision

#### Design and methodology

BioSystems Y15 Histamine method has been validated for seven matrixes: raw tuna, water-canned tuna, oil-canned tuna, raw salmon, raw sardines, oil-canned sardines, semi-preserved anchovy fillets. All matrixes were previously quantified with results <10 mg / kg of histamine by HPLC-UV/VIS method based on ISO 19343: 2017 HPLC, based on Duflos et al. method (9) in ANFACO-CECOPESCA (National Association of Manufacturers of Canned Fish and Seafood of Spain) laboratory. Samples were artificially spiked with concentrated aqueous solutions of histamine dihydrochloride (Sigma—H7250) to achieve a homogeneous distribution of the contaminant within the food batch. Each matrix was prepared and homogenized so that there were five known concentrations of histamine and zero (non-spiked), to cover the analytical range of the method (1.43–200 mg/kg range; test 0, 10, 50, 100, 150, and 200 mg/kg). Individual test portions where prepared as detailed in Table 4. It is considered that the added volume of stock spike solution is not significant for the calculation of concentration and was not taken into account.

**Table 4.** Details on preparation of contamination levels

Sample set	Level, mg/kg	Stock spike solution		Sample weight, g	Solution volume, µL
Sample set 1	0	N/A	N/A	5	N/A
Sample set 2	5	Solution 4	500 mg/L	5	50
Sample set 3	10	Solution 4	500 mg/L	5	10
Sample set 4	50	Solution 3	2000 mg/L	5	125
Sample set 5	100	Solution 2	5000 mg/L	5	100
Sample set 6	200	Solution 1	10000 mg/L	5	100

N/A: Not Applicable.

The test portions per concentration per matrix were randomized and blind coded. From each blinded sample, 5 g test portion were weighed out, extracted and analyzed by two analysts on two instruments over 9 days, one matrix each day according to the BioSystems Y15 Histamine method.

Linear regression was applied by plotting the determined concentration versus the spiked concentration. Goodness of fit (r^2^) was calculated.

#### Results

**Figure 10.**
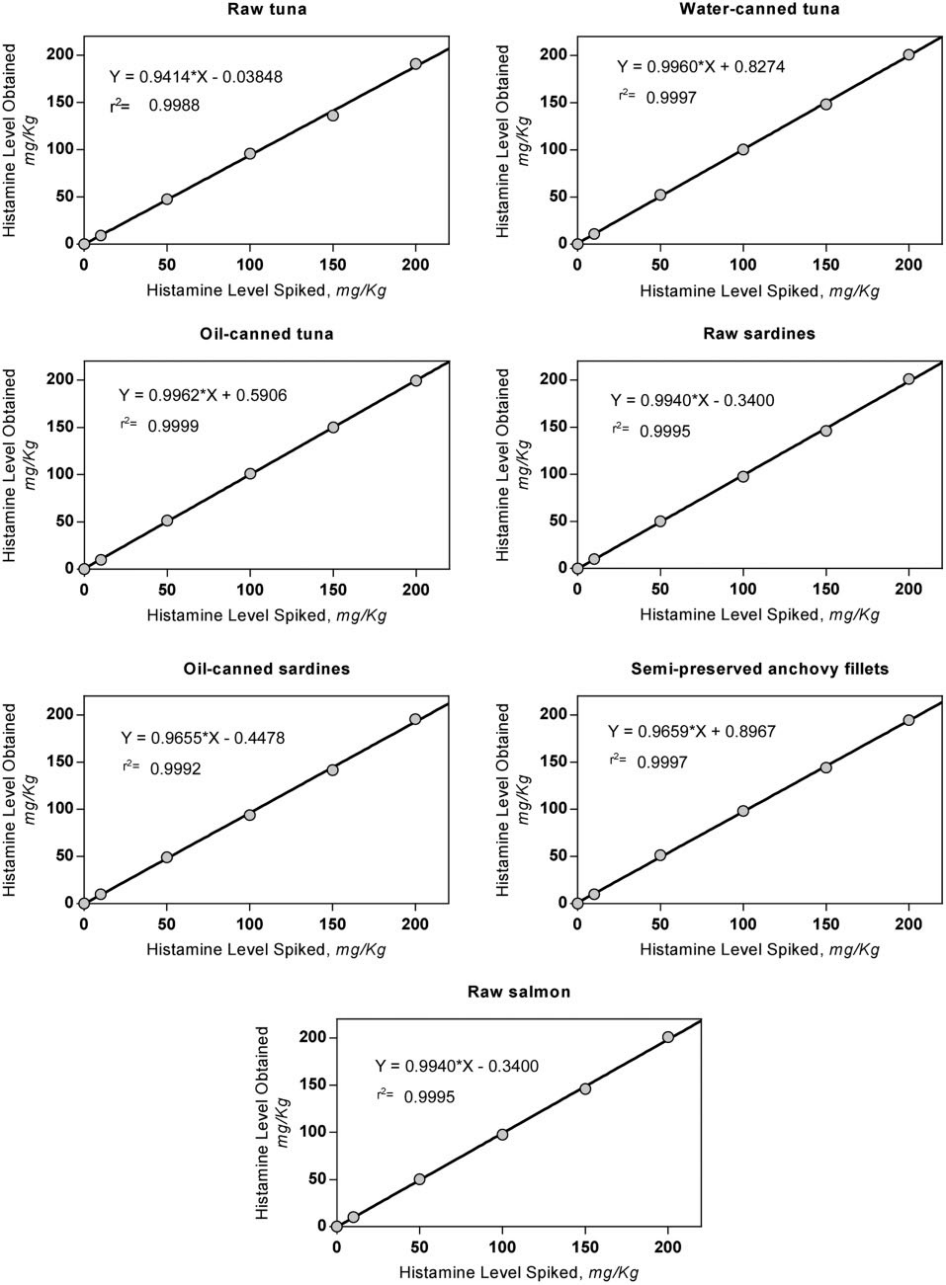
Method developer results plots for spiked matrixes.

The recovery obtained at all levels and with all the matrixes analyzed were within the acceptance range of 80–110% (Table 5). The lowest recovery (92%) corresponded to the addition of 10 mg/kg in raw tuna and the highest (107%) corresponded to the addition of 10 mg/kg in water-canned tuna, both within the acceptance criteria. The endogenous histamine content was included in the recovery calculations. The relative standard deviation of repeatability (RSD_r_) was determined for all matrixes and levels and in all cases values <10% are shown. All the matrixes showed an overall r^2^ exceeding 0.998 (Figure 10).

**Table 5.** Method developer results for spiked matrixes

Matrix	Naturally contaminated histamine, mg/kg	Spiking, mg/kg	Total histamine. mg/kg	BioSystems Y15 Histamine results				
				Mean (*n* = 5), mg/kg	S_r_	RSD_r_, %	Recovery, %	Bias, mg/kg
Raw tuna	1.2	0	1.2	1.2				
		10	11.2	10.3	0.09	7.81	92	–0.8
		50	51.2	48.9	0.33	3.16	96	–2.3
		100	101.2	97.0	1.94	3.96	96	–4.2
		150	151.2	147.5	2.74	2.83	98	–3.7
		200	201.2	192.1	6.93	5.04	95	–9.1
Water-canned tuna	2.1	0	2.1	2.1				
		10	12.1	12.9	0.10	4.78	107	0.8
		50	52.1	54.4	0.39	3.00	105	2.4
		100	102.1	102.4	1.11	2.04	100	0.3
		150	152.1	150.5	0.99	0.97	99	–1.6
		200	202.1	203.1	1.84	1.22	101	1.0
Oil-canned tuna	4.2	0	4.2	4.2				
		10	14.2	14.2	0.04	0.90	100	0.0
		50	54.2	55.6	0.18	1.26	103	1.4
		100	104.2	105.2	0.81	1.46	101	1.0
		150	154.2	154.0	1.13	1.07	100	–0.1
		200	204.2	203.5	1.39	0.90	100	–0.7
Raw sardines	5.1	0	5.1	5.1				
		10	15.1	15.2	0.25	4.95	101	0.1
		50	55.1	55.3	0.27	1.76	100	0.2
		100	105.1	102.6	1.43	2.59	98	–2.5
		150	155.1	151.2	1.83	1.78	98	–3.9
		200	205.1	206.1	1.38	0.91	101	1.0
Oil-canned sardines	5.0	0	5.0	5.0				
		10	15.0	14.8	0.19	3.83	99	–0.2
		50	55.0	54.0	0.28	1.89	98	–1.0
		100	105.0	98.7	0.54	1.00	94	–6.3
		150	155.0	146.6	1.37	1.39	95	–8.4
		200	205.0	200.7	1.99	1.36	98	–4.4
Semi-preserved anchovy fillets	3.3	0	3.3	3.3				
		10	13.3	13.1	0.14	4.26	98	–0.2
		50	53.3	54.7	0.45	3.44	103	1.4
		100	103.3	101.5	0.80	1.46	98	–1.8
		150	153.3	147.5	4.08	4.02	96	–5.8
		200	203.3	197.7	4.97	3.37	97	–5.6
Raw salmon	5.1	0	5.1	5.1				
		10	15.1	15.2	0.20	3.95	101	0.1
		50	55.1	55.3	0.32	2.10	100	0.2
		100	105.1	102.6	0.83	1.50	98	–2.5
		150	155.1	151.2	0.91	0.89	98	–3.9
		200	205.1	206.1	1.48	0.98	101	1.0

### Reference Method Comparison

#### Design and methodology

A series of samples of different types of matrixes with natural concentrations of histamine and with spiked histamine to obtain different concentrations along the measurement range were analyzed with a reference method at ANFACO-CECOPESCA (Vigo, Spain): HPLC-UV based on ISO 19343:2017 HPLC method (8) and with BioSystems Y15 Histamine method. Each sample was analyzed in duplicate by the two methods under repeatable conditions (Table 6).

**Table 6.** Results of comparison of the reference method and the BioSystems Y15 Histamine method

Sample	ANFACO HPLC, mg/kg	BioSystems Y15 Histamine, mg/kg
Raw mackerel	34	39
Raw mackerel (spiked)	61	64
Raw sardine	10	12
Raw sardine (spiked)	89	97
Pickled anchovy	437	450
Pickled anchovy (spiked)	592	567
Anchovy pate	<10 (4)	4
Raw tuna sirloin	<10 (0)	0
Raw tuna sirloin (spiked)	74	65
Oil-canned tuna	<10 (3)	2
Oil-canned tuna (spiked)	<10 (8)	8
Oil-canned tuna (spiked)	122	121
Oil-canned sardines	<10 (7)	7
Oil-canned sardines (spiked)	27	28
Oil-canned mackerel	<10 (0)	1
Oil-canned mackerel (spiked)	81	69
Raw tuna	<10 (1.1)	1.1
Water-canned tuna	<10 (5.5)	5.5
Oil-canned tuna	<10 (6.6)	6.5
Semi-preserved anchovy fillets	10.7	10.7

#### Results

**Figure 11.**
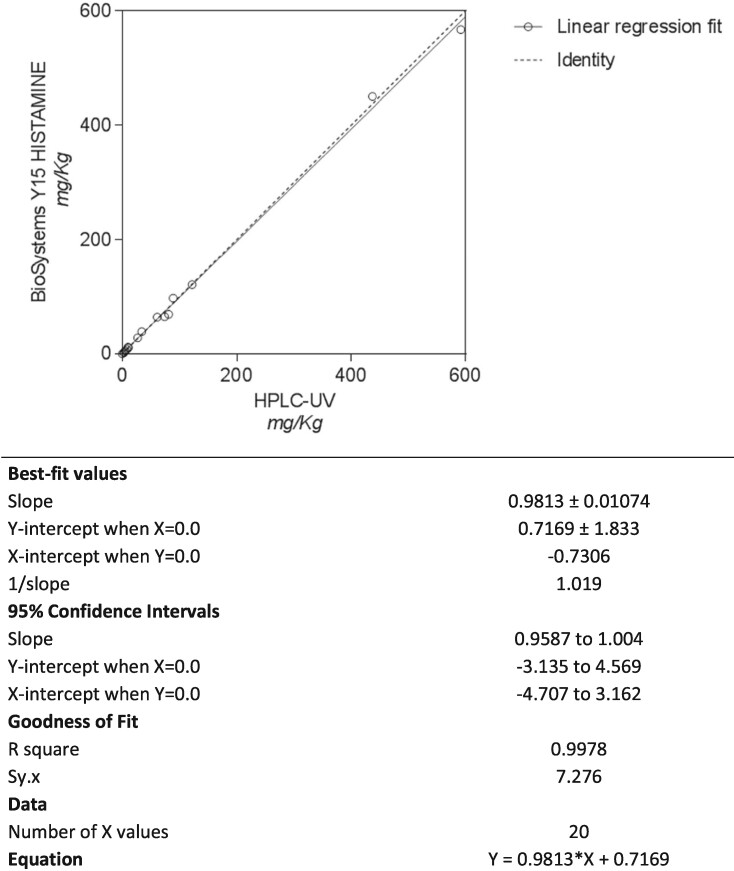
Graph and regression analysis data of HPLC-UV/VIS ISO 19343:2017 HPLC Duflos et al. method verssus BioSystems Y15 Histamine method in fresh fish and canned fish samples.

Out of the 20 analyzed samples, nine had concentrations below the LOQ of the HPLC method (<10 mg/kg). All these samples also gave results below 10 mg/kg with BioSystems Y15 Histamine method. The samples above the LOQ showed a very good correlation (r2 = 0.9978) by the linear regression analysis and no proportional or constant error was observed between the two methods. All statistics showed that both methods were equivalent (Figure 11).

### Reference Materials and Proficiency Tests

#### Design and methodology

A reference and a quality control material obtained from FAPAS were tested for several days by different technicians. The samples ranged from 16.6 to 216 mg/kg of histamine. BioSystems also participated in three rounds of the food chemistry proficiency test of canned fish organized by FAPAS (2016–2019). The samples ranged from 38.5 to 153 mg/kg. The samples covered the measuring range of the BioSystems Y15 Histamine method. Table 7 shows the type of sample, the matrix, number of participants, the percentage of results with a *z*-score of ≤2, the assigned value, and the results obtained by BioSystems.

**Table 7.** Reference materials and proficiency test results

Organizer	Type	Reference	Matrix		Total scores/ %|*Z*|≤ 2	Assigned value, mg/Kg	Result, mg/kg	*z*-Score	Ok?
FAPAS	Reference material	TET040RM	Canned fish	Pilchards in tomato sauce	N/A	16.6 (15,7–17,5)	16.4	N/A	YES
FAPAS	Quality control material	T27176QC	Canned fish	Not specified	N/A	216 (186–247)	204	N/A	YES
FAPAS	Food Chemistry Proficiency Test	27253	Canned fish	Tuna chunks in brine	157 / 73	38.5	43.6	1.4	YES
FAPAS	Food Chemistry Proficiency Test	27243	Canned fish	Tuna	116 / 71	128	138	1.0	YES
FAPAS	Food Chemistry Proficiency Test	27189	Canned fish	Pilchards in tomato sauce	85 / 73	153	160.3	0.6	YES

N/A: Not applicable.

#### N/A: Not applicable

 

#### Results

All the samples tested met the criteria established by FAPAS (reference materials within the concentration range and the proficiency test with results of *z*-score ≤2) demonstrating good accuracy and performance of the BioSystems Y15 Histamine method.

### LOD and LOQ Determination

#### Design and methodology

**Figure 12.**
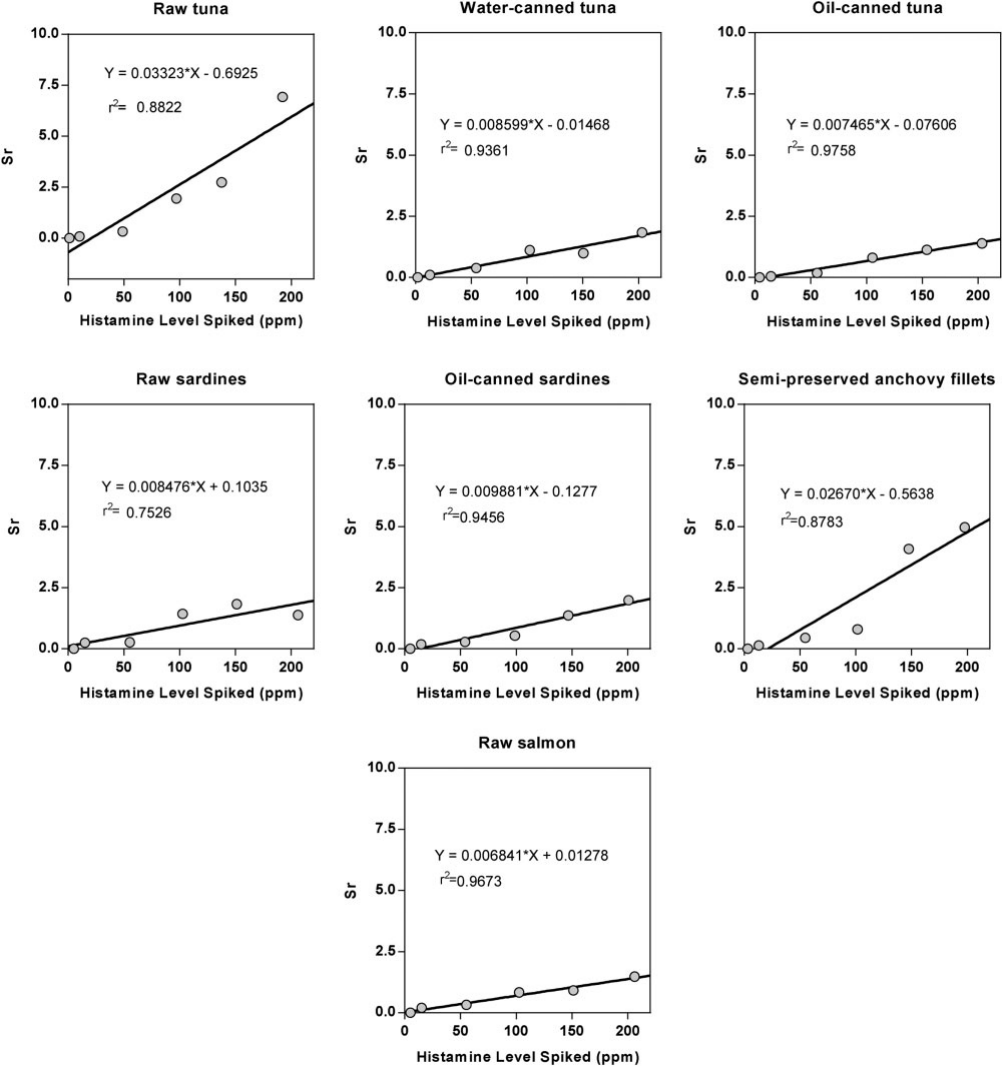
Method developer plots of Sr versus mean result for estimating LOD.

The LOD and LOQ were determined by plotting Sr against mean for the candidate method results of each matrix (Figure 12). LOD was calculated as:

$$\text{LOD}=\frac{{\bar{X}}_{0}+3.3(s_{b})}{1-1.65m}$$

where ${\bar{X}}_{0}$ = the mean analytical value of the no added histamine matrix, *S_b_* = the *y*-intercept of the line, and *m* = the slope of the line. The results of the LOD study are listed in Table 8. The LOQ was estimated as 3 × LOD and validated by spiking each matrix at or near the estimated LOQ and testing 10 replicates to demonstrate acceptable precision.

**Table 8.** Method developer estimation of LOD and LOQ

Detectability	Raw tuna	Water-canned tuna	Oil-canned tuna	Raw sardines	Oil-canned sardines	Semi-preserved anchovy fillets	Raw salmon
LOD, mg/kg	5.77	2.85	4.28	4.58	0.29	7.01	5.1
LOQ, mg/kg	17.0	8.5	13.0	14.0	0.87	21.0	15.3

#### Results

The estimates revealed LODs in the range from 0.29 to 7.01 mg/kg, whereas LOQs were estimated from 0.90 to 21 mg/kg. LOQ was verified in all cases at 10 mg/kg. To determine the influence of the matrix on the LOQ, 10 mg/Kg of histamine was added in each type of matrix. Acceptance criteria are a good precision with RSD_r_ of <10% for 10 replicates and recoveries in the range 80–110%. For all matrixes, an LOD of approximately 3 mg/kg and a LOQ of 10 mg/kg can be considered adequate. The data showed an RSD_r_ below 10% and recovery for all matrixes in the expected range. These results are presented in Table 9.

**Table 9:** Method developer LOQ study

Histamine	Raw tuna	Water-canned tuna	Oil-canned tuna	Raw sardines	Oil-canned sardines	Semi-preserved anchovy fillets	Raw salmon
Naturally contaminated sample, mg/kg	0.5	1.2	3.0	5.9	4.9	3.0	5.1
Spiked, mg/kg	+10.0	+10.0	+10.0	+10.0	+10.0	+10.0	+10.0
Measured value, mg/kg							
Replicate 1	9.0	11.1	12.4	17.3	14.6	12.8	15.3
Replicate 2	8.8	11.0	13.1	17.8	14.8	12.1	14.9
Replicate 3	9.6	11.0	12.9	16.4	14.8	11.8	15.1
Replicate 4	9.0	11.0	12.9	15.4	14.7	12.5	15.3
Replicate 5	9.4	11.3	12.7	15.9	14.5	11.9	14.8
Replicate 6	9.4	10.8	13.1	15.4	14.8	12.2	15.2
Replicate 7	9.1	10.9	12.6	15.7	14.6	12.6	15.4
Replicate 8	9.9	11.2	11.9	16.6	14.3	12.5	15.1
Replicate 9	8.8	10.8	12.6	16.1	14.6	11.9	15.2
Replicate 10	9.1	10.9	12.3	15.7	14.2	12.3	14.9
Mean, mg/kg	9.2	11.0	12.7	16.2	14.6	12.3	15.1
Recovery, %	88	98	97	102	98	94	100
S_r_	0.34	0.16	0.38	0.82	0.21	0.35	0.20
RSD_r_, %	3.65	1.47	3.02	5.05	1.41	2.82	1.32

### Product Consistency

#### Design and methodology

Each lot of histamine reagent manufactured at BioSystems is controlled based on established criteria to ensure lot-to-lot consistency. All these studies are carried out in different Y15 analyzers from the Quality Control department. The criteria used are:

- *Reagent blank.*—The absorbance obtained with a blank sample (water). This ensures that the chromogen used does not show large variations despite the fact that the raw material lot is different in each reagent lot.
- *Sensitivity.*—Ratio of the absorbance obtained with the calibrator 3 and the concentration of the same calibrator (10 mg/L). In this way we ensure the consistency of the absorbance regardless of the manufacturing process of each lot.
- *Accuracy.*—Performed with two internal controls of known concentration. The result is expressed as a ratio of the value obtained versus that expected. The acceptance criterion is ±10% with respect to the assigned value.

#### Results

The 13 lots studied met the established criteria and no significant statistical differences were found (Table 10).

**Table 10.** Lot-to-lot consistency^a^

	Reagent blank, A_420nm_^a^		Sensitivity, mA_420nm_·L/mg^b^			Accuracy ratio^c^			
Lot^d^	Measured	Tolerance	Measured	Tol. min.	Tol. max.	Control 1	Control 2	Tol. min.	Tol. max.
00001	0.056	<0.300	44.7	40.5	49.5	1.01	1.05	0.90	1.10
00004	0.055	<0.300	42.4	40.5	49.5	1.08	1.01	0.90	1.10
00014	0.065	<0.300	45.1	40.5	49.5	1.07	0.90	0.90	1.10
00026	0.065	<0.300	44.7	40.5	49.5	1.06	1.00	0.90	1.10
00029	0.068	<0.300	44.9	40.5	49.5	1.09	1.01	0.90	1.10
00031	0.073	<0300	46.2	40.5	49.5	1.04	1.00	0.90	1.10
00035	0.076	<0.300	45.6	40.5	49.5	1.05	0.99	0.90	1.10
00037	0.063	<0.300	44.9	40.5	49.5	1.07	1.01	0.90	1.10
00038	0.063	<0.300	46.4	40.5	49.5	1.04	0.98	0.90	1.10
00041	0.065	<0.300	45.2	40.5	49.5	1.07	1.03	0.90	1.10
00043	0.065	<0.300	43.7	40.5	49.5	1.09	1.05	0.90	1.10
00044	0.102	<0.300	43.2	40.5	49.5	1.09	1.04	0.90	1.10
00047	0.053	<0.300	44.9	40.5	49.5	1.02	0.99	0.90	1.10
Mean	0.067		44.8			1.06	1.00		
S_r_	0.012		1.110			0.027	0.038		
RSD_r_, %	18.6		2.48			2.50	3.82		

a Absorbance of the reagent blank at 420 nm.

b Ratio of the absorbance (in miliabsorbance) obtained with the calibrator 3 and the concentration of the same calibrator (10 mg/L).

c Ratio of the concentration (mg/L) obtained in the control of the reagent and the assigned concentration of the reference material (Control 1, 8 mg/L / Control 2, 13 mg/L).

d Results of several lots produced.

### Stability Study

#### Design and methodology

Reagents, once opened, were stored at the recommended storage temperature. At defined intervals and at the end of shelf-life the reagents were tested with the automated procedure using a BioSystems Y15.

#### Results

Results for the 24-month real time stability for the histamine reagent and standard kit are presented in Tables 11 and 12, where ratio is the relationship between the obtained and the target value. The results generated from all reagents were comparable, met the established criteria, and no significant statistical differences were found.

**Table 11.** Reagent real time stability

Month	Lot	Reagent blank, A_420nm_^a^	Sensitivity, mA_420nm_·L/mg^b^	Accuracy, ratio^c^
0	001	0.055	42.4	1.05
0	002	0.065	45.1	1.07
0	003	0.065	44.7	1.06
7	001	0.072	42.0	1.02
10	001	0.070	40.9	1.03
10	002	0.077	42.6	1.04
19	001	0.073	41.1	1.01
27	002	0.068	39.6	1.06
32	001	0.061	38.4	1.03
40	001	0.071	31.4	1.05

a absorbance of the reagent blank at 420 nm.

b Ratio of the absorbance (in milli-absorbance) obtained with the calibrator 3 by and the concentration of the same calibrator (10 mg/L).

c Ratio of the concentration (mg/L) obtained in the control of the reagent and the and the concentration (mg/L) of the reference material.

**Table 12.** Histamine standards real time stability

Standard	Lot	Months	Result	
			mg/L	ratio
Histamine S1	001PA	17	5.0	1.00
Histamine S1	004XA	18	5.1	1.02
Histamine S1	004XA	24	5.0	0.99
Histamine S1	001PA	28	5.0	1.00
Histamine S1	004XA	30	5.5	1.09
Histamine S1	001PA	36	4.9	0.97
Histamine S1	001PA	42	4.7	0.93
Histamine S5	001PA	17	19.5	0.97
Histamine S5	005XA	18	21.2	1.06
Histamine S5	005XA	24	20.2	1.01
Histamine S5	001PA	28	19.9	0.99
Histamine S5	005XA	30	21.0	1.05
Histamine S5	001PA	36	19.4	0.97
Histamine S5	001PA	42	19.9	1.00

### Robustness

#### Design and methodology

This study evaluates the ability of the method to remain unaffected by small variations in method parameters that might be expected to occur when the method is performed by an end user. These parameters are most likely to affect the analytical performance and determine the range of variation that can occur without adversely affecting the analytical results.

The following parameters were chosen: extraction volume (20, 25, and 30 mL), extraction time (10, 20, and 30 min), and storage time of the sample prior to analysis at room temperature (30 min, 1 h, and 2 h). The factorial design is translated into the Youden design (10) in Table 13 and the Youden-Steiner test was applied (Table 14). Treatment combination 9 shows the normal method parameter values. Treatment combinations 1–8 use either the high or low values for each parameter. For each treatment combination, two replicates of canned tuna in water spiked at 50 mg/kg histamine were analyzed.

**Table 13.** Youden-Steiner factorial design and results of robustness testing of water-canned tuna (spiked with 50 mg/kg histamine)

		Determination combination								
*F*-factor	Value of *F*-factor	1	2	3	4	5	6	7	8	9
Extraction volume	A: 20 mL	A	A	A	A	a	a	a	a	a′
	a: 30 mL								
	a′: 25 mL								
Extraction time	B: 10 min	B	B	b′	b′	B	B	b′	b′	b
	b: 20 min								
	b′: 30 min								
Sample storage prior to analysis, RT	C: 30 min	C	c′	C	c′	C	c′	C	c′	c
	c: 1 h								
	c′: 2 h								
BioSystems Y15 Histamine results, mg/Kg	Average data	48	47	47	49	50	52	48	48	48

**Table 14.** Youden-Steiner test results

Variable comparison (X-x)	Di^a^	SDi^b^	SDm^c^	Acceptance requirements	Complies?
Δ A, a	2.1	1.1	1.6	SDi≤SDm	YES
Δ A, a′	0.9	0.5			YES
Δ a, a′	1.6	0.8			YES
Δ B, b	1.9	1.0			YES
Δ b, b′	0.5	0.3			YES
Δ C, c	0.9	0.5			YES
Δ B, b′	2.0	1.0			YES
Δ C, c′	1.8	1.0			YES
Δ c, c′	1.5	0.8			YES

a Di = Average differences.

b SDi = Standard deviation of the differences.

c SDm = Standard deviation of the method (raw tuna 50 mg/kg).

#### Results

According to the Youden-Steiner test results, the method is considered robust because the standard deviation of the differences is, in all combinations, less than the standard deviation of the method at 50 mg/kg level in the water-canned tuna matrix evaluated.

## Independent Laboratory Studies

This validation outline evaluated the performance of the HDH test kit in three different fish samples (fresh tuna, water-canned tuna, oil-canned tuna). The matrix study has determined the bias, recovery, repeatability precision, LOD, and LOQ of the BioSystems Y15 Histamine method following the AOAC Guidelines. The reference method used was HPLC-UV (ISO 19343:2017) based on the Duflos et al. method(9). All analyses were performed at ANFACO-CECOPESCA (Vigo, Spain), and all the test kits as well as the test analyzer were provided by Biosystems S.A.

### Matrix Study: Accuracy and Precision

#### Design and methodology

The studies to determine the accuracy and precision performed in the independent laboratory (ANFACO) followed the same methodology described in the *Matrix Study: Accuracy and Precision* section within the *Method Developer* section with the following modifications: BioSystems Y15 Histamine method has been validated for three matrixes: raw tuna, water-canned tuna, oil-canned tuna, and each matrix was prepared and homogenized such that there were five known concentrations of histamine and zero (non-spiked), to cover the analytical range of the method (0, 5, 10, 50, 100, and 200 mg/kg).

Spiked samples and blanks were homogenized and prepared according to the protocol specified by the developer. All test portions were tested using the BioSystems Y15 analyzer, following the parameter setup in the software of the analyzer.

**Figure 13.**
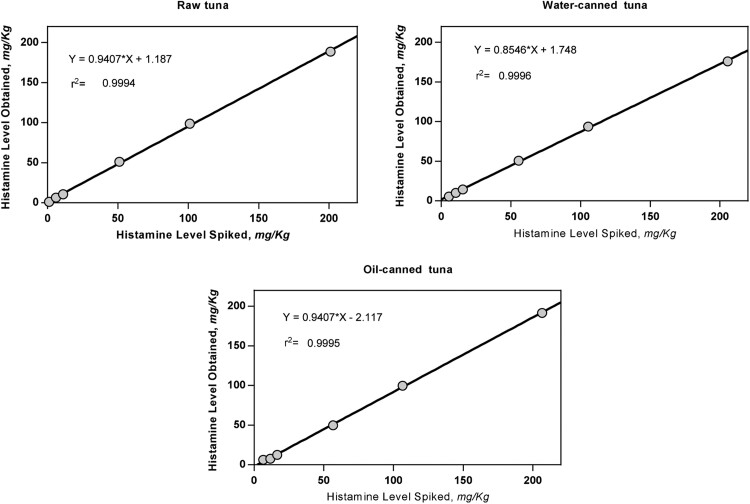
Independent laboratory results graph for spiked matrixes.

Linear regression was applied by plotting the determined concentration versus the spiked concentration and r^2^ was calculated (Figure 13).

#### Results

The recovery obtained at all levels and with all the matrixes analyzed are within the acceptance range of 80–110% (Table 15) except for oil-canned tuna sample spiked with 5 and 10 mg/kg of histamine, with a recovery of 66 and 75%, respectively. These results only occurred for one of the matrixes studied and were not reproduced in the study of the method developer. It may be due to the concentrations below the range of the method and very close to the LOQ. The endogenous histamine content was included in the recovery calculations. The RSD_r_ was determined for all matrixes and levels and ranged from 0.2 to 31.0%. If only the data corresponding to concentrations ≥10 mg/kg (LOQ) are considered, the RSD_r_ ranged from 0.2–6.0% demonstrating excellent precision in repeatability conditions. All the matrixes showed an overall r^2^ exceeding 0.999 (Figure 13).

**Table 15.** Independent laboratory results for spiked matrixes

Matrix	Endogenous histamine, mg/kg	Spiking, mg/kg	Total histamine mg/kg	BioSystems Y15 Histamine results				
				Mean (*n* = 5), mg/kg	S_r_	RSD_r_, %	Recovery, %	Bias, mg/kg
Raw tuna	1.1	0	1.1	1.1	0.3	31.0	100	0
		5	6.1	6.3	0.60	10.0	104	0.2
		10	11.1	10.6	0.60	6.0	96	–0.5
		50	51.1	51.2	0.90	1.8	100	0.1
		100	101.1	98.8	0.20	0.2	98	–2.3
		200	201.1	188.7	1.80	1.0	94	–12.3
Water-canned tuna	5.5	0	5.5	5.5	0.30	5.0	100	0
		5	10.5	10.1	0.20	1.9	96	–0.4
		10	15.5	14.4	0.50	3.4	93	–1.1
		50	55.5	50.8	0.70	1.3	92	–4.7
		100	105.5	93.7	2.60	2.7	89	–11.8
		200	205.5	176.1	1.10	0.7	86	–29.4
Oil-canned tuna	6.6	0	6.6	6.5	0.20	3.1	98	–0.1
		5	11.6	7.6	0.30	4.2	66	–3.9
		10	16.6	12.4	0.70	5.5	75	–4.1
		50	56.6	49.7	0.50	0.9	88	–6.8
		100	106.6	99.9	0.80	0.8	94	–6.6
		200	206.6	191.8	2.70	1.4	93	–14.7

### LOD and LOQ Determination

#### Design and methodology

**Figure 14.**
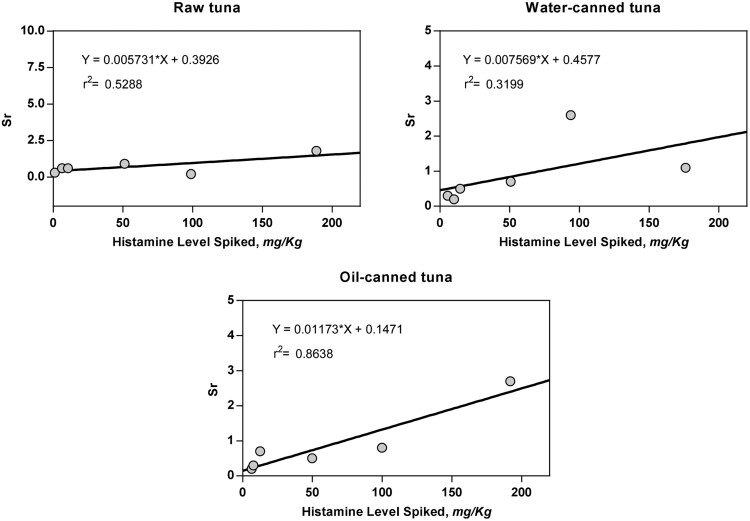
Independent laboratory plots of Sr versus mean result for estimating LOD.

The studies to determine the accuracy and precision performed in the independent laboratory (ANFACO) followed the same methodology described in the *Matrix Study: Accuracy and Precision* section within the *Method Developer* section. The results of the LOD study are listed in Table 16. The LOQ was estimated as 3 × LOD and validated by spiking each matrix at or near the estimated LOQ and testing 10 replicates to demonstrate acceptable precision. These results are presented in Table 17 and Figure 14.

**Table 16.** Independent laboratory estimation of LOD and LOQ

Detectability	Raw tuna, mg/kg	Water-canned tuna, mg/kg	Oil-canned tuna, mg/kg
LOD	2.41	6.99	7.03
LOQ	7.22	20.98	21.09

**Table 17.** Independent laboratory LOQ study

Histamine	Raw tuna	Water-canned tuna	Oil-canned tuna
Naturally contaminated sample, mg/kg	1.10	5.50	6.50
Spiked, mg/kg	+10.00	+20.00	+20.00
Measured value, mg/kg			
Replicate 1	11.68	22.65	23.57
Replicate 2	11.20	23.72	23.14
Replicate 3	11.37	23.26	22.39
Replicate 4	11.90	23.15	23.10
Replicate 5	11.39	23.71	23.19
Replicate 6	11.87	23.01	23.74
Replicate 7	11.51	23.15	23.28
Replicate 8	11.30	22.61	23.08
Replicate 9	12.27	23.20	23.08
Replicate 10	12.70	22.98	23.53
Mean, mg/kg	11.72	23.14	23.21
Recovery, %	106	91	88
S_r_	0.48	0.37	0.37
RSD_r_, %	4.06	1.61	1.60

#### Results

The estimates revealed LODs in the range from 2.42 to 7.12 mg/kg, whereas LOQs were estimated from 7.26 to 21.37 mg/kg. The LOQs were verified by spiking the raw tuna matrix at 10 mg/kg and the water and oil-canned tuna at 20 mg/kg to investigate the influence of each claimed matrix. Acceptance criteria are a good precision with RSD_r_ of <10% for 10 replicates and recoveries in the range 80–110%. The RSD_r_ for 10 mg/kg spiked raw tuna was <5% whereas the RSD_r_ for 20 mg/kg spiked water and oil-canned tuna was <2%. The data shows RSD_r_ below 10% and recovery for all matrixes is in the expected range.

## Discussion

The BioSystems Y15 Histamine method evaluated in this validation following the protocols established by the AOAC is applicable for the quantification of histamine in samples of raw fish and canned fish. Automation of the measurement with an analyzer allows measurements to be obtained quickly, easily, and with high precision, accuracy, and robustness since user intervention is minimized upon extraction. The extraction protocol compared to other methods (HPLC, Fluorometry, or ELISA) has been shown to be simple, fast, and does not require hazardous solvents since it is done with water.

The method developer validation included linearity, selectivity, and interference studies, recovery, accuracy, precision, comparison to reference methods for fishery products, proficiency tests data, estimates of LOD and LOQ, matrix-specific confirmation of LOQ, robustness studies, lot-to-lot consistency, and stability testing for reagent and standards.

Linearity in the measurement range (0–200 mg/kg) has been confirmed according to the regression statistics. This range is adequate to be able to quantify whether or not the fish samples comply with current legislations and with the quality criteria. The measuring range can be increased according to users’ needs by diluting samples made automatically by the analyzer.

Of the 11 substances similar to histamine used in the selectivity study, only positive interference with agmatine was observed. This interference is considered very insignificant since it would have relevance only in cases in which the ratio between histamine and agmatine was very low and the fish is unlikely to contain high concentrations of agmatine. Nevertheless, the BioSystems Y15 Histamine instructions for use supplied with each kit, warns the user that in the presence of agmatine, there may be positive interferences.

Recovery studies showed excellent results with all types of matrixes studied throughout the entire sample range and even with histamine concentrations below the quantification limit of the method. These results were confirmed in the comparison study against the accredited reference method based on HPLC-UC/VIS conducted by ANFACO. The data showed a very good correlation with the ISO 19343:2017 HPLC method based on the Duflos et al. method. Good accuracy was also confirmed using FAPAS control and reference materials and from the participation in proficiency test schemes from FAPAS, all with z-scores of ≤2.

The repeatability data are very good (at 50 mg/kg the worst case is 3.44% in the semi-preserved anchovy matrix and the best 1.26% in oil-canned tuna). This data can be obtained thanks to the ease of the extraction protocol and the little intervention of the user in the handling of the reagents and in the measurement protocol.

The study of the LOD/LOQ estimates was performed according to the basis of blank samples and the LOQ was confirmed by spiking experiments. According to the data obtained, an LOQ of 10 mg/kg was established. In the verification carried out with the repeatability study, with real samples spiked with 10 mg/kg of histamine, RSD_r_ <5% and recoveries close to 100% were obtained in all cases, indicating that the LOQ of 10 mg/kg is valid and it could even be lower.

The stability testing of 13 independent lots showed high lot-to-lot reproducibility and that the control carried out during the manufacture of the kit components ensures that there are no differences in results regardless of the lot used. Stability studies of both reagent and calibrators proved that kits are stable over the claimed shelf life of two years from the manufacturing date.

A thorough robustness testing scheme was performed. None of the conditions that were altered with respect to the protocol described caused significant variations in the result, demonstrating that the BioSystems Y15 Histamine method is robust.

An evaluation of the BioSystems Y15 Histamine was performed by an independent laboratory and consisted of the analysis of spiked fresh raw and canned fish, including precision, recovery, and verification of LOQ. The data obtained revealed that the kit works with the same precision in minimally trained hands as with expert method developers. The recovery was very similar to that obtained by the method developer. The estimated LOQ obtained for the raw tuna sample was equivalent to that obtained by the method developer, but for water and oil-canned tuna, a higher estimated LOD was obtained which generates an LOQ of 20 mg/kg. In the validation of the LOQ, by spiking each matrix at or near the estimated LOQ and testing 10 replicates to demonstrate acceptable precision, very low RSDr were obtained: <5% for raw tuna (10 mg/kg) and <2% for the two samples of canned fish (20 mg/kg). The recovery is within the established tolerances (80–110%). These data suggest that the LOQ could have been overestimated.

.

## Conclusions

The BioSystems Y15 Histamine method offers a fast, accurate, and automated determination of histamine in various fish matrixes and other fishery products. Sample preparation is quick, simple, and does not use hazardous solvents. The automation of the measurement in the BioSystems Y15 analyzer improves precision and accuracy, reduces the potential for user errors, and allows flexibility since it is possible to analyze a few samples or up to 150 samples/hour. The BioSystems Y15 Histamine method offers high ease of use and robustness facilitating its use by non-expert users ensuring high quality standards in results.
